# Trends of Liver Stiffness in Inflammatory Bowel Disease with Chronic Hepatitis C

**DOI:** 10.3390/diagnostics10121037

**Published:** 2020-12-02

**Authors:** Giuseppe Losurdo, Andrea Iannone, Antonella Contaldo, Michele Barone, Enzo Ierardi, Alfredo Di Leo, Mariabeatrice Principi

**Affiliations:** 1 Section of Gastroenterology, Department of Emergency and Organ Transplantation, University of Bari, 70124 Bari, Italy; giuseppelos@alice.it (G.L.); ianan@hotmail.it (A.I.); contaldoantonella@gmail.com (A.C.); michele.barone@uniba.it (M.B.); ierardi.enzo@gmail.com (E.I.); b.principi@gmail.com (M.P.); 2 Ph.D. Course in Organs and Tissues Transplantation and Cellular Therapies, Department of Emergency and Organ Transplantation, University “Aldo Moro” of Bari, 70124 Bari, Italy

**Keywords:** hepatitis C, inflammatory bowel disease, direct anti viral agents, liver stiffness, elastography

## Abstract

Concomitant inflammatory bowel disease (IBD) and hepatitis C virus (HCV) infection is a relevant comorbidity since IBD itself exposes to a high risk of liver damage. We aimed to evaluate liver stiffness (LS) in IBD-HCV after antiviral treatment. We enrolled IBD patients with HCV. All patients at baseline underwent LS measurement by elastography. Patients who were eligible for antiviral therapy received direct antiviral agents (DAAs) and sustained viral response was evaluated at the 12th week. A control group was selected within IBD patients without HCV. One year later, all IBD-HCV patients and controls repeated LS measurement. Twenty-four IBD-HCV patients and 24 IBD controls entered the study. Only twelve out of 24 received DAAs and all achieved sustained viral response (SVR). All IBD subjects were in remission at enrollment and maintained remission for one year. After one year, IBD patients who eradicated HCV passed from a liver stiffness of 8.5 ± 6.2 kPa to 7.1 ± 3.9, *p* = 0.13. IBD patients who did not eradicate HCV worsened liver stiffness: from 7.6 ± 4.4 to 8.6 ± 4.6, *p* = 0.01. In the IBD control group, stiffness decreased from 7.8 ± 4.4 to 6.0 ± 3.1, *p* < 0.001. In conclusion, HCV eradication is able to stop the evolution of liver fibrosis in IBD, while failure to treat may lead to its progression. A stable IBD remission may improve LS even in non-infected subjects.

## 1. Introduction

Inflammatory bowel disease (IBD) is a chronic inflammatory condition with autoimmune pathogenesis affecting the digestive tract. It encompasses two main clinical entities, i.e., ulcerative colitis (UC) and Crohn’s disease [1,2]. IBD is often associated to other comorbidities. Concomitant chronic hepatitis C (CHC) has been described in several cohorts of IBD patients. Chen et al. [3] found a HCV prevalence of 1% in ulcerative colitis, similar to controls. On the other hand, Longo et al. [4] found a seroprevalence of 5.98%. This value was similar to that reported in a recent experience of our group, i.e., 3.4% [5]. Hepatitis C virus (HCV) infects worldwide about 177 million people, and it is one the most diffuse causes of cirrhosis [6]. It has been postulated that concomitant IBD-CHC is a relevant comorbidity since IBD itself exposes to high risk of liver damage due to drugs assumed to treat IBD, such as azathioprine, for the possible association with primary sclerosing cholangitis, and the increased risk of liver steatosis in IBD [7,8,9]. For these reasons, some authors have underlined that the evolution of fibrosis in IBD patients may be faster. Indeed, in the paper by Loras et al. [10], it was shown that double immunosuppression increased the risk of virus reactivation and worsened liver functioning by 8.75 times. Therefore, the treatment of HCV is of primary importance in order to stop the natural history of liver fibrosis. In the past, the use of interferon-based treatments has set a series of questions in the field of IBD about its potential ability to stimulate immune system in subjects with autoimmune disorders. Indeed, despite there being several lines of evidence describing its safety [11,12,13], some reports showed the onset or the relapse of IBD during interferon therapy [14,15,16]. Beside side effects and the low success rate (about 50%), interferon has been advised with caution in IBD and with suboptimal diffusion. However, with the advent of novel direct antiviral agents (DAAs), which have few side effects and a sustained viral response (SVR) close to 100%, the natural history of CHC has radically changed [17], and novel opportunities for IBD-CHC patients have appeared.

Therefore, we performed a prospective study aiming to evaluate the evolution of liver fibrosis in IBD patients with HCV (Table S1). In detail, some of them received DAA therapy, others did not. All patients were followed for one year to evaluate the variations of liver stiffness by elastography.

## 2. Materials and Methods

### 2.1. Patients Selection and Protocol

The study was approved by the independent Ethics Committee of the Policlinico di Bari (protocol no. 4862, 3 May 2017) and was performed according to the Helsinki declaration 1975 statements. We enrolled IBD patients with CHC, revealed by positive serum detection of anti-HCV antibodies and HCV-RNA, in the period October 2016–October 2018. All patients underwent anti-HCV antibody evaluation and, in the case of positivity, HCV-RNA dosing and genotype analysis were performed. All patients at baseline underwent measurement of liver stiffness by elastography. Then, those patients who were eligible for antiviral therapy according to the Italian indications of the “Agenzia Italiana del Farmaco”, received DAA. Therefore, we followed up HCV treated and untreated subjects. One year later, all IBD-HCV patients had a second measurement of liver stiffness.

**Figure 1:**
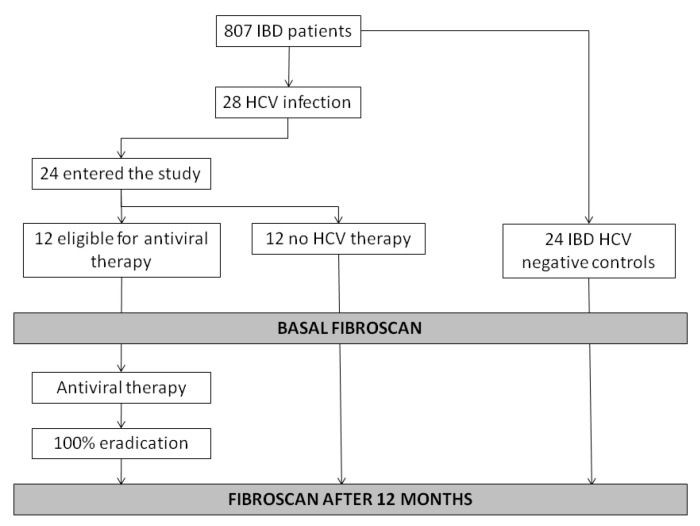
Flowchart reporting patients selection and study protocol. IBD: inflammatory bowel disease; HCV: hepatitis C virus.

A control group was selected within IBD patients without CHC, matched for age, sex, IBD characteristics and therapies, comorbidities, liver function and fibrosis. Analogously, the control group had a liver elastography at baseline and after one year (Figure 1).

For each patient the following data were recorded: age, sex, disease extension and behavior according to Montreal classification [18], IBD specific therapy, liver function tests, anthropometric measures.

For HCV patients, the genotype and viral load (HCV-RNA) were assayed before starting DAA. The DAA was chosen according to the “Agenzia Italiana del Farmaco” and European Association for the Study of the Liver (EASL) guideline indications [19]. If sustained viral response (undetectable HCV-RNA) at 12th week post-treatment was achieved, HCV was considered as eradicated.

Patients who did not receive treatment during the follow up of this study received a further chance to be treated after the end of the study if eligibility criteria were met.

### 2.2. Evaluation of Hepatic Fibrosis Stage Using Fibroscan

Each patient, fasting for at least 12 h, underwent the determination of liver stiffness by Fibroscan (Echosens, Paris, France) at the time of enrollment and one year later. Measurements of the liver stiffness were performed on the right lobe of the liver, on a site localized by previous ultrasound evaluation, through the intercostal spaces with patient in supine position. Ten valid measurements were performed on each patient and only exams with a success rate of at least 60% and an interquartile interval <30% were considered reliable [20]. We excluded from the study conditions that technically prevented the execution of liver elastography (severe obesity, narrowness of intercostal spaces). Cut-off for different degrees of fibrosis were chosen according to the literature [21]. All operators had performed at least 50 examinations according to the recent statement, which considers Fibroscan as a procedure with a rapid learning curve and that is very reliable [21].

### 2.3. Statistical Analysis

Continuous variables were expressed as mean ± standard deviation or median and interquartile range (IQR), while categorical variables as percentages. The comparison between continuous variables was performed by Student’s *t* test (or Mann–Whitney U in case of non-Gaussian distribution), while the chi-square test was used for categorical variables. Before–after analysis of continuous variables was performed by the Wilcoxon signed rank test (due to the non-parametric distribution after the Kolmogorov–Smirnov test). All statistical tests were two-tailed and statistical significance was set at *p* < 0.05. The statistical analysis was performed using the software SPSS version 23.0 for Windows, Armonk, NY: IBM Corp and GraphPad Prism version 5.00 for Windows (GraphPad Software, San Diego, CA, USA).

## 3. Results

### 3.1. Baseline Features

Among a database of 807 IBD patients, 28 tested positive for HCV. Of these, 24 accepted to participate to the study (Figure 1). Their mean age was 58.5 ± 14.9 years, and the male/female ratio was 1:1. Seventeen (70.8%) had Crohn’s disease (CD) and seven (29.2%) ulcerative colitis (UC). CD patients had, in most of cases, an ileal involvement (41.2%) with a non-stenosing, non-penetrating pattern (58.8%). The 71.4% of UC patients had proctitis. Nine IBD-HCV patients experienced almost a biologic therapy (four infliximab, four adalimumab, one golimumab). All patients were in clinical remission, as demonstrated by the partial Mayo and HBI in Table 1. The more common HCV genotypes were: 1b in 13 patients (54.2%), and two in six subjects (25%).

Twenty-four IBD patients without HCV composed the control group. As detailed in Table 1, they had similar features for age, sex, IBD characteristics and therapy and laboratory investigations. Both groups had a comparable basal stiffness (8.1 ± 5.3 kPa for IBD-HCV and 7.8 ± 4.4 for controls-IBD, *p* = 0.86). Similarly, most of patients had an F0 fibrosis at Fibroscan (66.7% in IBD-HCV and 53.2% in IBD-controls, *p* = 0.54), while F4 was detected in 16.6% of HCV-IBD and in 12.5% of IBD-controls (Table 2). The three patients with F4 without HCV had metabolic syndrome. An ultrasound appearance of steatosis was found in 33.3% of HCV-IBD and in 45.8% of IBD-controls (*p* = 0.55).

None of patients in both groups had any further cause of chronic liver disease (primary biliary cholangitis, primary sclerosing cholangitis, alcoholic liver disease).

### 3.2. HCV Treatment and Liver Fibrosis Evolution

Only 12 out of 24 HCV-IBD patients underwent DAA therapy. Some patients, despite being eligible to DAAs, decided to postpone therapy and entered the non-eradicated HCV-IBD group.

**Figure 2:**
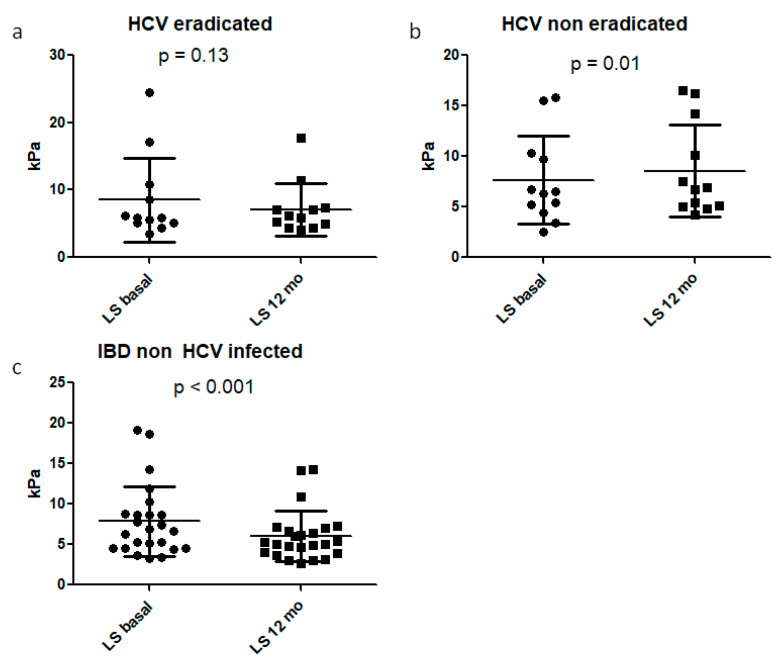
Variations in liver stiffness from baseline to 12 months in IBD patients who eradicated (**a**) or non eradicated HCV (**b**), as well as in IBD control group (**c**).

Six patients with genotype 1b received sofosbuvir/ledipasvir (five patients) or ombitasvir/paritaprevir/ritonavir plus dasabuvir (one patient). Four patients with genotype two received sofosbuvir/velpatasvir. One patient with genotype three had sofosbuvir/daclatasvir. One patient with genotype four had sofosbuvir/velpatasvir. The duration was 12 weeks in all subjects. All HCV subjects achieved SVR12 and no adverse event was recorded. Twelve months after therapy, all patients repeated Fibroscan. All patients, both HCV infected and controls, were still in IBD clinical remission phase and did not need to change therapy. IBD patients who eradicated HCV showed non-significant changes of liver stiffness values, from 8.5 ± 6.2 (median 5.8 IQR 5.125–10.23) to 7.1 ± 3.9 (median 6.05, IQR 4.45–7.325), with a *p* = 0.13 (see Figure 2a).

IBD patients who did not eradicate HCV showed significantly worsened values of liver stiffness: from 7.6 ± 4.4 (median 6.4 IQR 4.6–10.15) to 8.6 ± 4.6 (median 6.8 IQR 5.025–13.18), with a *p* = 0.01, as illustrated in Figure 2b.

In the IBD control group, liver stiffness decreased from 7.8 ± 4.4 (median 6.75 IQR 4.5–8.775) to 6.0 ± 3.1 (median 5.15 IQR 3.925–6.9), with a significant improvement (*p* < 0.001), as shown in Figure 2c.

Regarding the stage of liver fibrosis, in the 12 HCV eradicated patients the stage was stable in eight patients, while we recorded three improvements (one from F1 to F0, one from F3 to F1 and one from F4 to F3), while one case of progression (from F0 to F1) was observed.

In the 12 patients who did not eradicate HCV, fibrosis was stable in 10, while in two patients a worsening was noted (one from F2 to F4 and one from F0 to F1). None improved fibrosis stage.

Among the 24 IBD patients without hepatitis, 18 had a stable stage. No progression of fibrosis was observed, while in six cases we found an improvement (two from F1 to F0, two from F3 to F0, one from F4 to F3 and one from F2 to F0).

**Figure 3:**
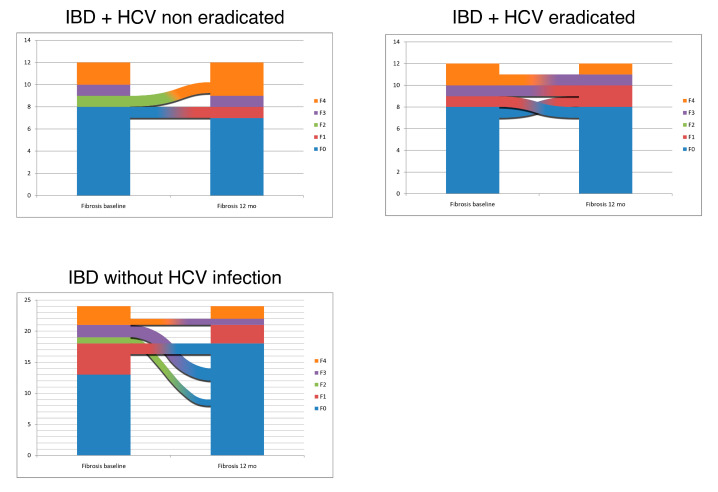
Variations in fibrosis severity stage across the 12 months follow up in the three groups of IBD patients.

All the variations in fibrosis stage are graphically represented in Figure 3.

## 4. Discussion

The advent of novel DAAs has dramatically improved the global picture of HCV infection. One of the most important effects is the reduction in the need of transplantation for cirrhotic patients with HCV [22]. Resolution of chronic HCV infection has shown, in several studies, that the regression of fibrosis measured by liver elastography can be achieved [23,24]. Treating HCV in IBD patients is an urgent need in order to stop the evolution of fibrosis in a double chronicity setting, which is of particular interest if we consider that some drugs such as thiopurines may be hepatotoxic.

In the present study, we aimed to evaluate liver stiffness in IBD-HCV patients after antiviral treatment. In particular, 24 IBD-HCV patients and 24 IBD controls were recruited. Twelve out of 24 received DAAs and all achieved SVR. After one year, IBD patients who eradicated HCV showed decreased values of stiffness (from 8.5 ± 6.2 kPa to 7.1 ± 3.9, *p* = 0.13). IBD patients who did not eradicate HCV achieved a worsened liver stiffness: from 7.6 ± 4.4 to 8.6 ± 4.6, *p* = 0.01. Interestingly, even in the IBD control group, a decreased stiffness from 7.8 ± 4.4 to 6.0 ± 3.1, (*p* < 0.001) was observed.

To the best of our knowledge, few case reports have been published about effective treatment of HCV by DAA in IBD patients [25,26]. Therefore, this is the first report on the topic on a well-defined series of patients. We demonstrated that DAAs in the IBD setting is very effective (SVR12 of 100%) without relevant adverse events. It should be underlined that novel DAAs do not show any potentially harmful interaction with IBD drugs, except for methotrexate [27]; however, none of our patients assumed such molecule. Moreover, another aspect of the present study that is worthy of mentioning, is the one-year follow up to evaluate the evolution of liver stiffness. Results showed that the eradication allowed a stabilization of fibrosis, while IBD patients who did not treat HCV encountered a worsening of fibrosis. This finding strongly supports the need for treating all IBD-HCV patients. Another intriguing result is the improvement of liver stiffness in our control group, constituted by IBD without HCV. Since all patients were in remission phase and retained such condition for the entire follow up, it is arguable that a prolonged disease inactivity may mirror a positive effect on liver fibrosis. The regression of stiffness occurring even in the control group could apparently underpower the effect of viral eradication. However, it should be noted that liver histological abnormalities (inflammation, fibrosis) were noted even in early seminal papers on IBD, and such alterations were correlated with the severity and long-standing disease [28,29]. Therefore, the improvement of fibrosis may take place after adequate disease control in patients with other autoimmune intestinal diseases such as in celiac disease-related hepatic damage [30].

It may be claimed that one year is a too short a time to verify fibrosis regression, however, it has been demonstrated that the magnitude of liver stiffness decline is greater in the first year after DAAs, while it tends to plateau from 1 year onwards [24]. A longer follow up could provide more interesting results, and this is under evaluation in our center for a future investigation. Another note of caution concerns the patients who did not eradicated HCV. Indeed, after the follow up, novel indications from “Agenzia Italiana del Farmaco”allowed a universal strategy of HCV treatment, therefore they were all eligible for treatment, and received a further chance to be treated. The results in this subgroup are under evaluation since they may strengthen the importance of our findings.

Additionally, it is important to remark that we did not find any correlation between drugs used for IBD and fibrosis variation, therefore our study was not biased by the possible interference of hepatotoxic drugs like azathioprine. Moreover, the control group was comparable with IBD-HCV for drugs and disease features, and this is another factor limiting bias. Additionally, it has been suggested that liver transaminase elevation may lead to an overestimation of liver fibrosis by Fibroscan, as recently highlighted [31,32]. However, in our cohort, in most of cases AST and ALT levels were within normal range or slightly increased, as highlighted in Table 2, and therefore, we may be confident that in our patients, stiffness was not overestimated

Our report highlights the importance of HCV eradication in IBD patients. In this setting, several strategies have been proposed when immunosuppression is necessary in such patients [27,33]. The sequential strategy (induction of IBD remission at first and then treatment with DAAs) has been recognized as the most reasonable. A concomitant strategy, consisting of contemporary administration of DAAs and immunosuppressive agents, is considered safe as well, but a drug interaction check is mandatory. Finally, inverse sequential strategy (DAA administration before biologics) has not been investigated yet, but it is theoretically promising, since it would allow to eradicate HCV before immunosuppression. In this regard, an interesting report concerns three patients who received DAAs and maintained SVR at 48 months despite a strong immunosuppression by anti-cancer drugs [34].

The low number of enrolled patients could be considered as a limitation for this study. However, the frequency of both comorbidities is quite uncommon, and the HCV-IBD population was drawn from a much larger IBD cohort of more than 800 subjects attending our outpatient unit.

## 5. Conclusions

HCV eradication is able to stop the evolution of liver fibrosis in IBD, while failure to treat it may lead to its aggravation. On the other hand, a stable IBD remission could improve liver stiffness and, most importantly, the kinetics of liver fibrosis is quite fast and the effects may be observed even in a one year period.

## Figures and Tables

**Table 1:** Comparison of demographic, anthropometric and metabolic characteristics of patients with IBD with or without HCV.

Variabile	IBD with HCV (n = 24)	IBD without HCV (n = 24)	*p* Value
Age, years (mean ± SD)	58.5 ± 14.9	51.1 ± 14.5	0.09
Male sex, n (%)	12 (50)	14 (58.3)	0.77
Smokers, n (%)	1 (4.2)	3 (12.5)	0.61
Diabetes, n (%)	2 (8.3)	2 (8.3)	1
Hypertension (%)	8 (33.3)	2 (8.3)	0.07
Waist circumference * n (%)	6 (25)	5 (20.8)	1
BMI, Kg/m^2^ (mean ± SD)	24.5 ± 3.2	24.2 ± 3.3	0.75
Metabolic syndrome, n (%)	4 (16.6)	3 (12.5)	1
Total cholesterol (mg/dL) (mean ± SD)	174.1 ± 36.4	172.2 ± 34.9	0.88
HDL (mg/dL) (mean ± SD)	72.2 ± 48.9	57.8 ± 13.5	0.52
LDL (mg/dL) (mean ± SD)	87.0 ± 19.5	97.1 ± 25.1	0.48
Triglycerides (mg/dL) (mean ± SD)	91.5 ± 18.9	117.6 ± 59	0.23
Fasting blood glucose (mg/dL) (mean ± SD)	95.5 ± 35.7	90.1 ± 11.6	0.55
Crohn’s disease, n (%)	17 (70.8)	12 (50)	0.07
HBI (mean ± SD)	0.4 ± 0.5	0.3 ± 0.4	0.48
CD behavior, n (%) B1 inflammatory B2 stenosing B3 penetrating p Perianal	10 (58.8) 4 (23.5) 3 (17.7) 0 (0)	4 (33.3) 4 (33.3) 4 (33.3) 0 (0)	0.35
CD localization n (%) L1 Ileal L2 Colon L3 Ileocolic L4 Upper GI	7 (41.2) 4 (23.5) 5 (29.4) 1 (5.9)	5 (41.7) 3 (25) 4 (33.3) 0 (0)	0.86
Previous surgery for CD, n (%)	2 (11.8)	2 (16.7)	1
Ulcerative colitis, n (%)	7 (29.2)	12 (50)	0.07
Mayo score (mean ± SD)	1.7 ± 1.2	1.2 ± 0.9	0.40
UC localization, n (%) E1 Proctitis E2 left colitis E3 subtotal/pancolitis	5 (71.4) 0 (0) 2 (28.6)	7 (58.3) 2 (16.7) 3 (25)	0.44
Azathioprine, n (%)	5 (20.8)	8 (33.3)	0.52
Systemic steroids, n (%) <3 courses/year >3 courses/year	14 (58.3) 1 (4.2)	13 (54.2) 3 (12.5)	0.58
Topical steroids, n (%) In course Used previously	5 (20.8) 3 (12.5)	13 (54.2) 3 (12.5)	0.54
Infliximab, n (%)	4 (16.7)	5 (20.8)	1
Adalimumab, n (%)	4 (16.7)	4 (16.7)	1
Golimumab, n (%)	1 (4.2)	1 (4.2)	1
Antibiotics, n (%)	14 (58.3)	13 (54.2)	1
Probiotics, n (%)	16 (66.7)	14 (58.3)	0.79
ESR (mm/h) (mean ± SD)	12.3 ± 6.9	16.8 ± 16.4	0.31
CRP (mg/dL) (mean ± SD)	5.3 ± 14.4	1.5 ± 1.4	0.22

IBD: inflammatory bowel diseases; BMI: body mass index; HCV: hepatitis C virus; ULN: beyond the upper normality limit; HDL: high density lipoprotein; LDL: low density lipoprotein; UC: ulcerative colitis; CD: Crohn’s disease; HBI: Harvey Bradshaw Index; ESR: erythrocyte sedimentation rate; CRP: C reactive protein; * subjects with a value >102 cm for males and >88 cm for females.

**Table 2:** Comparison of characteristics of liver profile in patients with IBD with or without HCV at baseline.

Variabile	IBD with HCV (n = 24)	IBD without HCV (n = 24)	*p* Value
Ethanol assumption, n (%) <10 g/day	2 (8.3)	4 (16.7)	0.67
Ultrasound liver steatosis, n (%)	8 (33.3)	11 (45.8)	0.55
Basal Liver stiffness, kPa (mean ± SD)	8.1 ± 5.3	7.8 ± 4.4	0.86
Severity of fibrosis, n (%) • F0 • F1 • F2 • F3 • F4	16 (66.7) 1 (4.2) 1 (4.2) 2 (8.3) 4 (16.6)	13 (54.2) 5 (20.8) 1 (4.2) 2 (8.3) 3 (12.5)	0.54
HCV genotype • **1a** • **1b** • **2** • **3** • **4**	1 (4.2) 13 (54.2) 6 (25) 2 (8.3) 2 (8.3)	-	-
AST (x ULN)	0.78 ± 0.47	1.1 ± 2.2	0.52
ALT (x ULN)	0.70 ± 0.55	0.68 ± 0.27	0.85
GGT (x ULN)	0.50 ± 0.34	0.79 ± 1.16	0.34
ALP (x ULN)	0.98 ± 1.29	0.90 ± 1.18	0.86
Total bilirubin (mg/dL) (mean ± SD)	0.83 ± 0.69	0.96 ± 0.95	0.63
Conjugated bilirubin (mg/dL) (mean ± SD)	0.26 ± 0.14	0.23 ± 0.21	0.63
Platelets (×10^3^) (mean ± SD)	246 ± 73	246 ± 115	0.99

IBD: inflammatory bowel diseases; AST: aspartate transaminase; ULN: beyond the upper normality limit; ALT: alanine transaminase; GGT: gamma glutamyl transpeptidase; ALP: alkaline phosphatase; APRI: AST to Platelet Ratio Index.

## Supplementary Materials

The following are available online at https://www.mdpi.com/2075-4418/10/12/1037/s1, Table S1: STROBE Statement—checklist of items that should be included in reports of observational studies.
